# Supplementary material for: Molecular mechanism of Mad1 kinetochore targeting by phosphorylated Bub1
Source: EMBO Rep. 2021 May 19;22(7):e52242. doi: 10.15252/embr.202052242 (PMC8391104; doi:10.15252/embr.202052242)
Supplement: Supplementary file 1 — Appendix [file EMBR-22-e52242-s001.pdf]

Appendix for

Molecular Mechanism of Mad1 Kinetochore Targeting by Phosphorylated Bub1

Elyse Fischer[1], Conny W.H. Yu[1], Dom Bellini[1], Stephen H. McLaughlin[1], Christian Orr[2], Armin Wagner[2], Stefan M.V. Freund[1] and David Barford[1,3]

[1]MRC Laboratory of Molecular Biology, Cambridge Biomedical Campus, Francis Crick Avenue, Cambridge, CB2 0QH, UK

[2]Diamond Light Source Ltd, Diamond House, Harwell Science & Innovation Campus, Didcot, Oxfordshire, OX11 0DE, UK

[3]Correspondence: dbarford@mrc-lmb.cam.ac.uk

Table of Contents

Appendix Table S1: Data collection and refinement statistics. **Pg. 2**

Appendix Table S2: Anomalous Data Statistics **Pg. 3**

Appendix Fig S1: Titration of Bub1^{CD1} peptide into Mad1^{CTD} **Pg. 4**

Appendix Fig S2: Secondary structure prediction of Bub1 and sequence conservation of Mad1^{CTD} **Pg. 5**

Appendix Fig S3: Phosphate SAD of the Bub1^{CD1}-Mad1^{CTD} complex **Pg. 6**

Appendix Fig S4: Isothermal calorimetry of Mad1 and Bub1 **Pg. 7**

Appendix Fig S5: Isothermal calorimetry of Mad1^{CTD} RLK mutants **Pg. 8**

Appendix Fig S6: Isothermal calorimetry of Mad1^{CTD} head domain mutants **Pg. 9**

Appendix Fig S7: Backbone resonances of Mad1^{CTD} RLK motif residues **Pg. 10**

Appendix Fig S8: Backbone resonances of Mad1^{CTD} head domain residues **Pg. 11**

Appendix Fig S9: Alignments of apo and bound Mad1^{CTD} **Pg. 12-13**

Appendix Table S1. Data collection and refinement statistics.

	**Mad1^{CTD}-Bub1^{CD1} ($P2_12_12_1$)**	**Mad1^{CTD}-Bub1^{CD1} ($P2_1$)**	**Mad1^{CTD}-Bub1^{CD1} ($P2_12_12$)**
**PDB ID**	7B1F	7B1H	7B1J
**Data Collection**	DLS i04	DLS i04	DLS i04
Wavelength (Å)	0.9795	0.9795	0.9795
Resolution range (Å)	40.25 - 1.75 (1.81 - 1.75)	39.43 - 2.4 (2.49 - 2.4)	34.76 - 2.90 (3.00 - 2.90)
Space group	$P2_12_12_1$	$P2_1$	$P2_12_12$
Unit cell (Å)	34.2 80.5 134.0 90.0° 90.0° 90.0°	34.63 132.34 82.75 90.0° 93.34° 90.0°	87.68 133.98 34.75 90.0° 90.0° 90.0°
Dimers per asymmetric unit	1	2	1
Total reflections	191826 (2738)	100346 (4893)	62788 (2992)
Unique reflections	33937 (1863)	28972 (2901)	9638 (454)
Multiplicity	5.6 (3.2)	3.5 (3.4)	3.6 (3.6)
Completeness (%)	88.7 (44.5)	99.6 (99.0)	100 (99.6)
Mean I/sigma(I)	16 (0.25)	14.5 (1.7)	25.9 (3.9)
Wilson B-factor (Å^2)	30	43	89.9
R-merge	0.05(1.97)	0.06 (0.78)	0.03 (0.47)
R-meas	0.06 (2.3)	0.07 (0.93)	0.04 (0.51)
R-pim	0.02 (1.2)	0.04 (0.50)	0.01 (0.20)
CC1/2	1.0 (0.30)	1.0 (0.68)	1.0 (.98)
**Refinement**			
Reflections used in refinement (N)	32886 (1294)	28946 (2898)	9541 (892)
Reflections used for R-free (N)	1569 (68)	1376 (110)	480 (47)
R-work	0.2558	0.2314	0.2618
R-free	0.2873	0.2806	0.2972
Non-hydrogen atoms (N)	2408	4542	2262
Protein residues (N)	282	565	282
RMS (bonds) (Å)	0.013	0.014	0.024
RMS (angles) (°)	1.52	1.90	2.33
Ramachandran favoured (%)	98.85	96.49	92.88
Ramachandran allowed (%)	1.15	3.33	6.37
Ramachandran outliers (%)	0.00	0.18	0.75
Rotamer outliers (%)	0.81	0.80	1.59
Molprobity Score	1.81	2.07	2.70
Average B-factor (Å^2)	56.95	61.78	104.52
Subunits (N)	4	8	4

Statistics for the highest-resolution shell are shown in parentheses.

Appendix Table S2: Anomalous Data Statistics

	**i23 Data**
**Data Collection**	DLS i23
Wavelength (Å)	2.7552
Resolution range (Å)	131 - 2.4 (2.49 - 2.40)
Space group	$P2_12_12_1$
Unit cell (Å)	34.58 84.56 131.89<br>90.0° 90.0° 90.0°
Dimers per asymmetric unit	1
Total reflections (N)	585078 (50188)
Unique reflections (N)	15780 (1601)
Multiplicity	37.1 (31.3)
Completeness (%)	99.4 (97.6)
Mean I/sigma(I)	29.5 (2.6)
Wilson B-factor (Å^2)	61
R-merge	0.078 (1.545)
R-meas	0.080 (1.595)
R-pim	0.013 (0.282)
CC1/2	1.0 (0.899)
Anomalous completeness (%)	99.1 (96.6)
Anomalous multiplicity	19.9 (16.6)
Mid slope	1.258

Statistics for the highest-resolution shell are shown in parentheses.

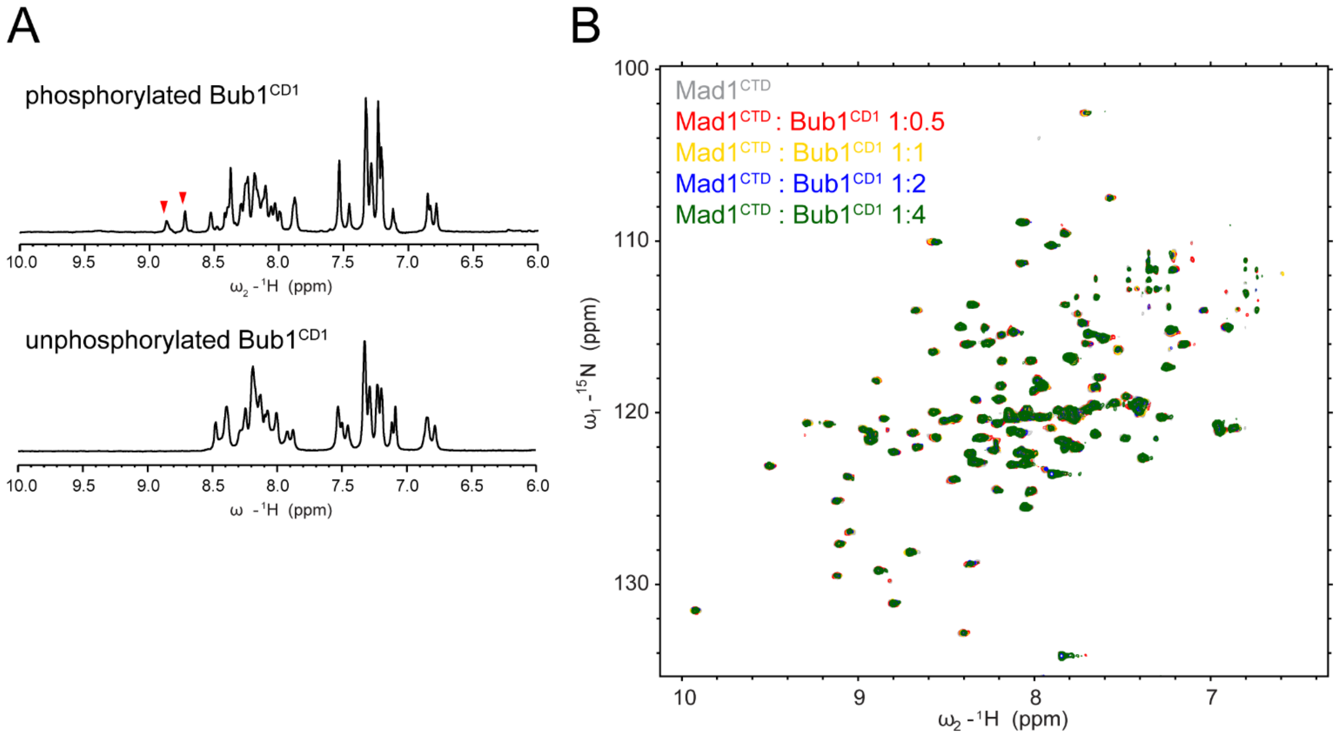

Appendix Fig S1: Titration of Bub1^{CD1} peptide into Mad1^{CTD}.

A ^{1}H 1D spectra showing the amide regions of phosphorylated and unphosphorylated Bub1^{CD1} peptides. The narrow dispersion of the amide peaks suggests both peptides are unstructured. The red arrows highlight two peaks that were shifted downfield upon phosphorylation, most likely corresponding to the phosphorylated Ser459 and Thr461.

B ^{1}H,^{15}N-2D HSQC showing ^{15}N-labelled Mad1^{CTD} with an increasing concentration of unphosphorylated Bub1^{CD1} peptide. In molar ratios of Mad1^{CTD} dimer to Bub1^{CD1}, peptides were added at 1:0.5 (red), 1:1 (yellow), 1:2 (blue) and 1:4 (green) ratios. There is no observable change in chemical shifts or peak intensities.

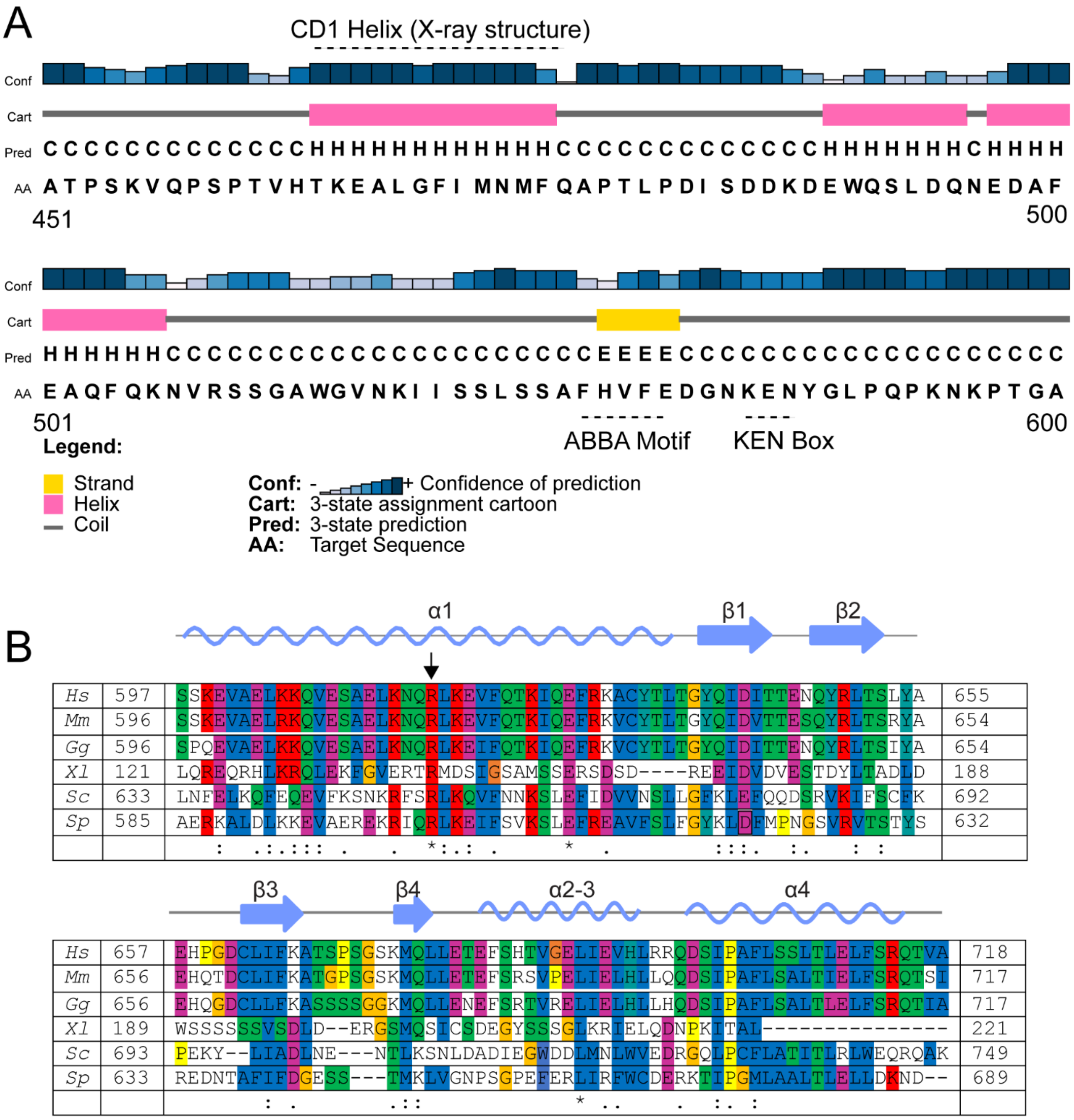

Appendix Fig S2: Secondary structure prediction of Bub1 and sequence conservation of Mad1^{CTD}.

A Secondary structure prediction by PSIPRED of Human Bub1 (residues 448-553) which includes the CD1, ABBA, and KEN1 domains of Bub1 (Buchan & Jones, 2019). The length of the CD1 helix present in the X-ray structure is shown as well as the location of the ABBA and KEN1 motifs.

B Sequence alignment created using ClustalX2 of Mad1^{CTD} (human residues 597-718) (Larkin *et al*, 2007). The secondary structural elements are shown above and the Arg617 residue of the RLK motif is highlighted with a black arrow.

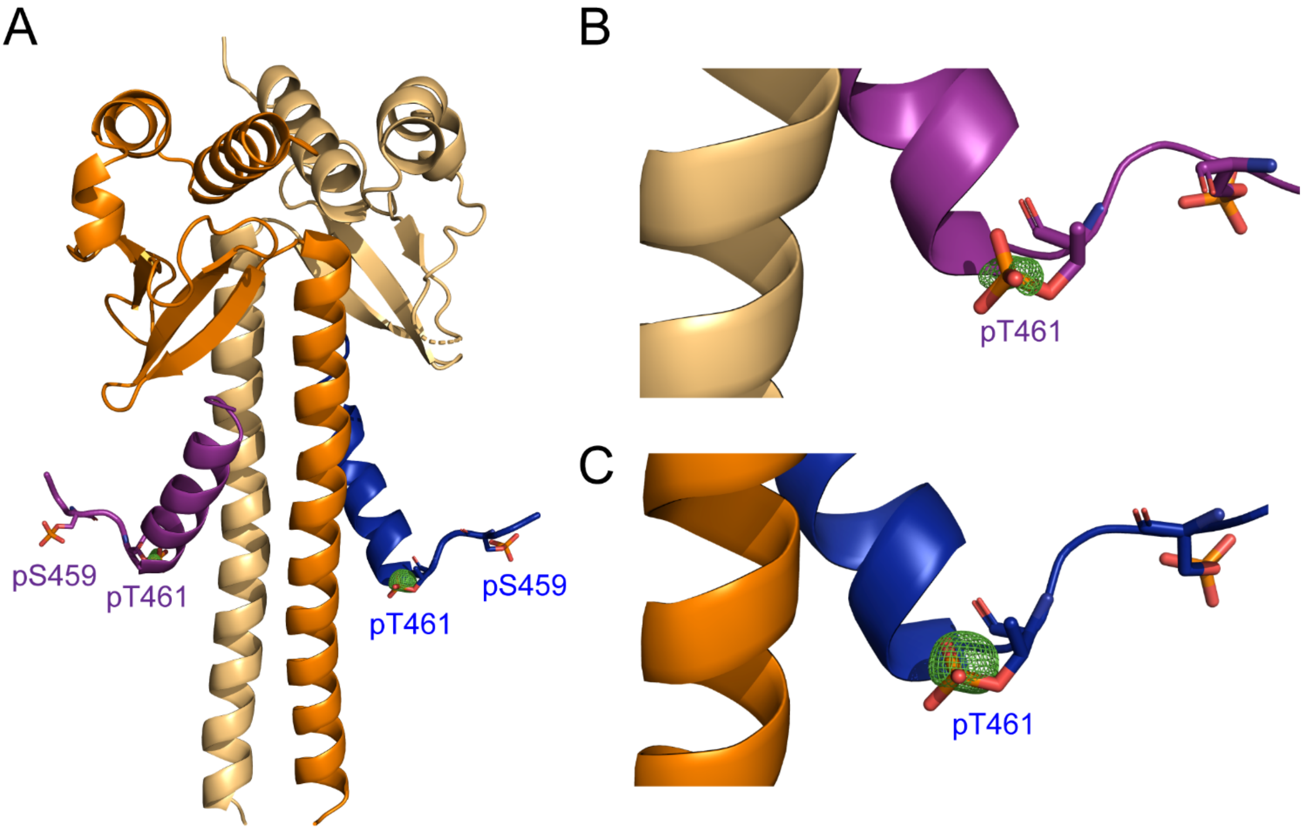

D

Nearest Atom	Distance to closest atom (Å)	Anomalous peak height (sig)
SG A:CYS65	0.605	15.30
SD A:MET78	1.040	10.24
SG B:CYS65	0.880	7.17
SG B:CYS36	0.481	6.95
CE B:MET78	0.824	6.25
SG A:CYS36	0.782	5.67
O B:GLY63	2.226	5.18
CB A:CYS36	1.876	5.14
O2P C:TPO4	1.165	5.06
O2P D:TPO4	1.595	4.82
CG D:MET17	2.352	4.45
OG1 D:TPO4	0.847	4.42
CG C:MET17	0.713	4.41
CA A:THR27	1.213	4.40
O2P C:TPO4	2.544	4.26
CG2 A:VAL24	2.315	4.17

Appendix Fig S3: Phosphate SAD of the Bub1^{CD1}-Mad1^{CTD} complex.

- A Crystal structure of Bub1^{CD1}-Mad1^{CTD} with the anomalous signal for the phosphate groups of the two phosphorylated threonine residues shown as a green mesh.
- B Close-up view of the phosphothreonine anomalous signal on subunit D.
- C Close-up view of the phosphothreonine anomalous signal on subunit C.
- D The strongest unique anomalous peaks as reported by ANODE with a cut off of 4.0 sigma (Thorn & Sheldrick, 2011). The anomalous peak heights corresponding to the phosphate of the phosphothreonine are highlighted in red.

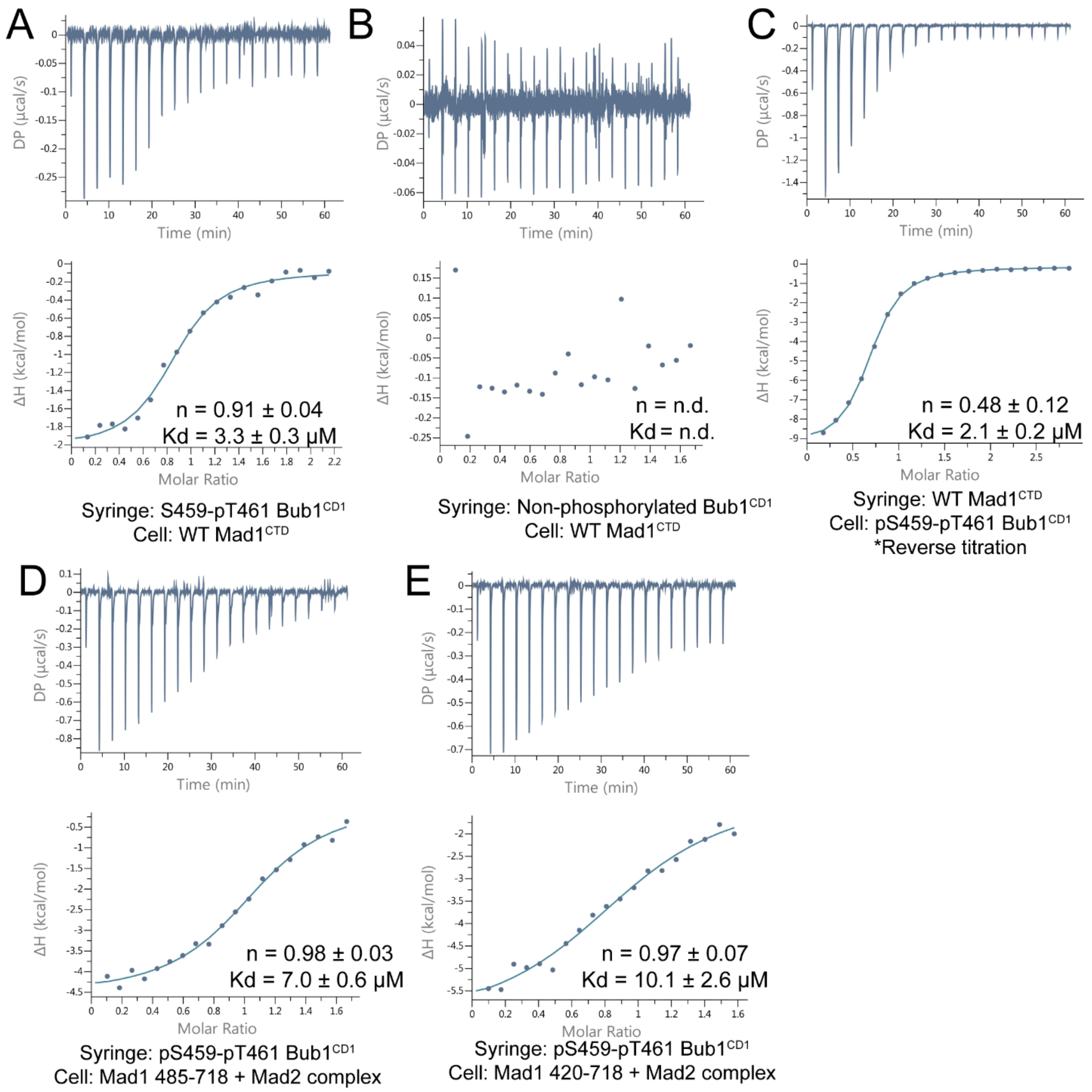

Appendix Fig S4: Isothermal calorimetry of Mad1 and Bub1

The K_D and stoichiometry (n) values were obtained by averaging at least three experiments. The reported error values are calculated standard deviations. Mad1^{CTD} was in the cell and the Bub1^{CD1} peptide was in the syringe, unless otherwise noted.

- A Titration of a singly phosphorylated pThr461 Bub1^{CD1} peptide to Mad1^{CTD}.
- B Titration of non-phosphorylated Bub1^{CD1} to Mad1^{CTD}.
- C Titration of Mad1^{CTD} to doubly phosphorylated pThr461-pSer459 Bub1^{CD1} where Mad1^{CTD} concentration is stated as total monomer.
- D Titration of doubly phosphorylated pThr461-pSer459 Bub1^{CD1} to the Mad1:C-Mad2 tetramer using Mad1 residues 485-718.
- E Titration of doubly phosphorylated pThr461-pSer459 Bub1^{CD1} to the Mad1:C-Mad2 tetramer using Mad1 residues 420-718.

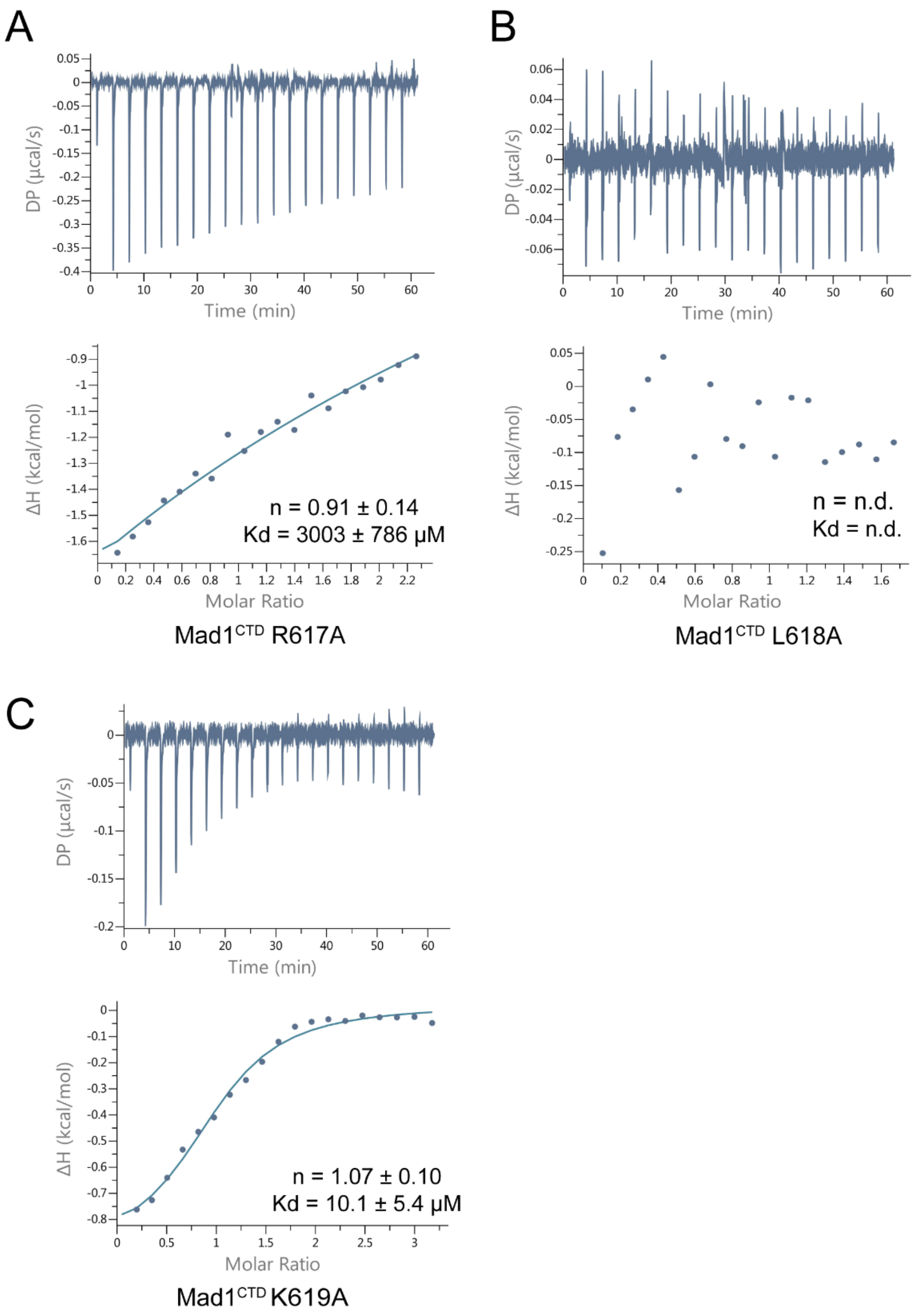

Appendix Fig S5: Isothermal calorimetry of Mad1^{CTD} RLK mutants

The K_D and stoichiometry (n) values were obtained by averaging at least three experiments. The reported error values are calculated standard deviations. Mad1^{CTD} was in the cell and Bub1^{CD1} peptide was in the syringe, unless otherwise noted.

- A Titration of doubly phosphorylated pThr461-pSer459 Bub1^{CD1} peptide to Mad1^{CTD} R617A RLK mutant.
- B Titration of doubly phosphorylated pThr461-pSer459 Bub1^{CD1} peptide to Mad1^{CTD} L618A RLK mutant.
- C Titration of doubly phosphorylated pThr461-pSer459 Bub1^{CD1} peptide to Mad1^{CTD} K619A RLK mutant

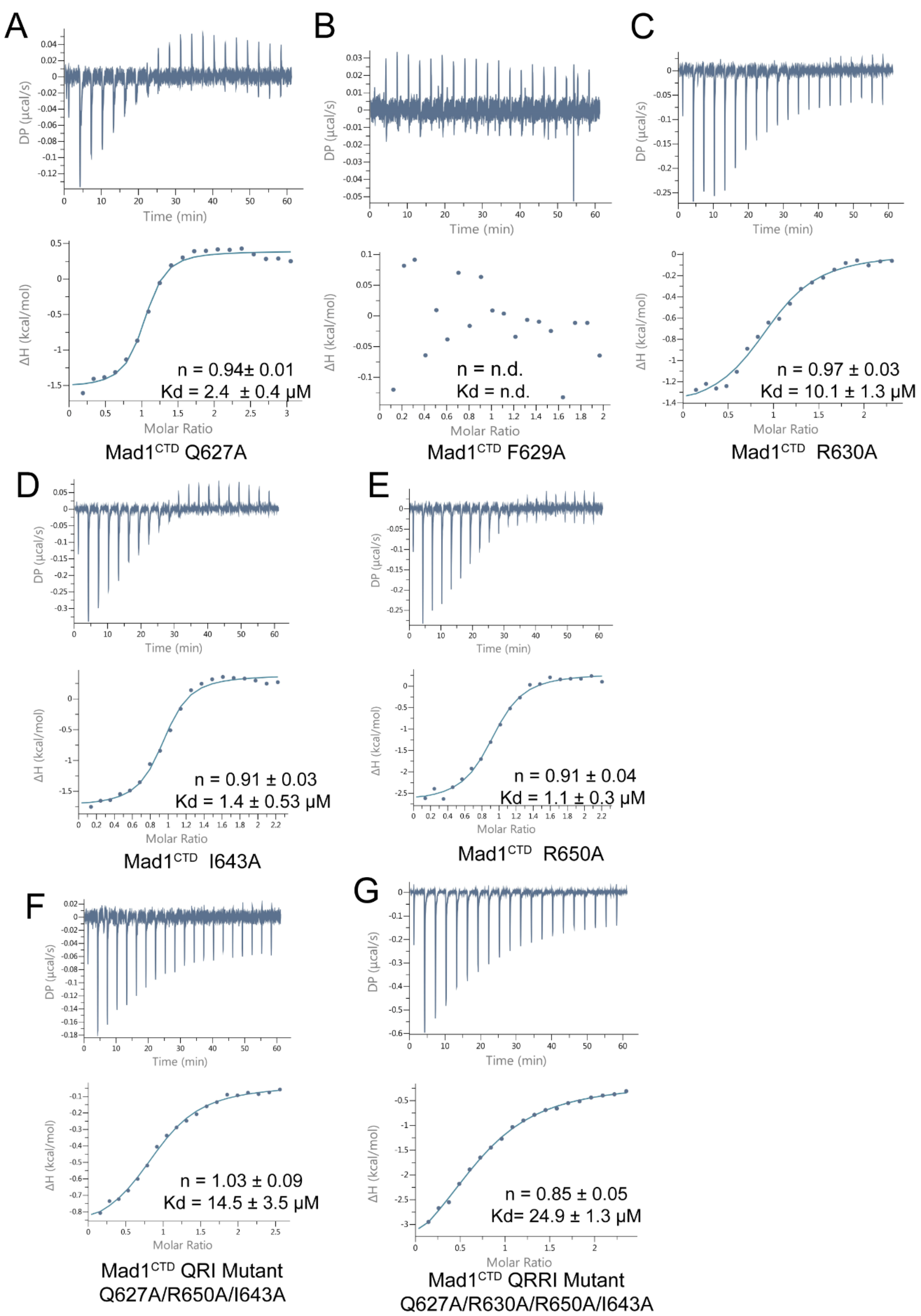

Appendix Fig S6: Isothermal calorimetry of Mad1^{CTD} head domain mutants
The Mad1^{CTD} head domain mutants tested are highlighted within the structure in Fig 2B. In A-G, the kD and stoichiometry (n) values were obtained by averaging at least three experiments. The reported error values are calculated standard deviations. In each case, the Mad1^{CTD} mutant was in the cell and a doubly phosphorylated pThr461-pSer459 Bub1^{CD1} peptide was in the syringe.

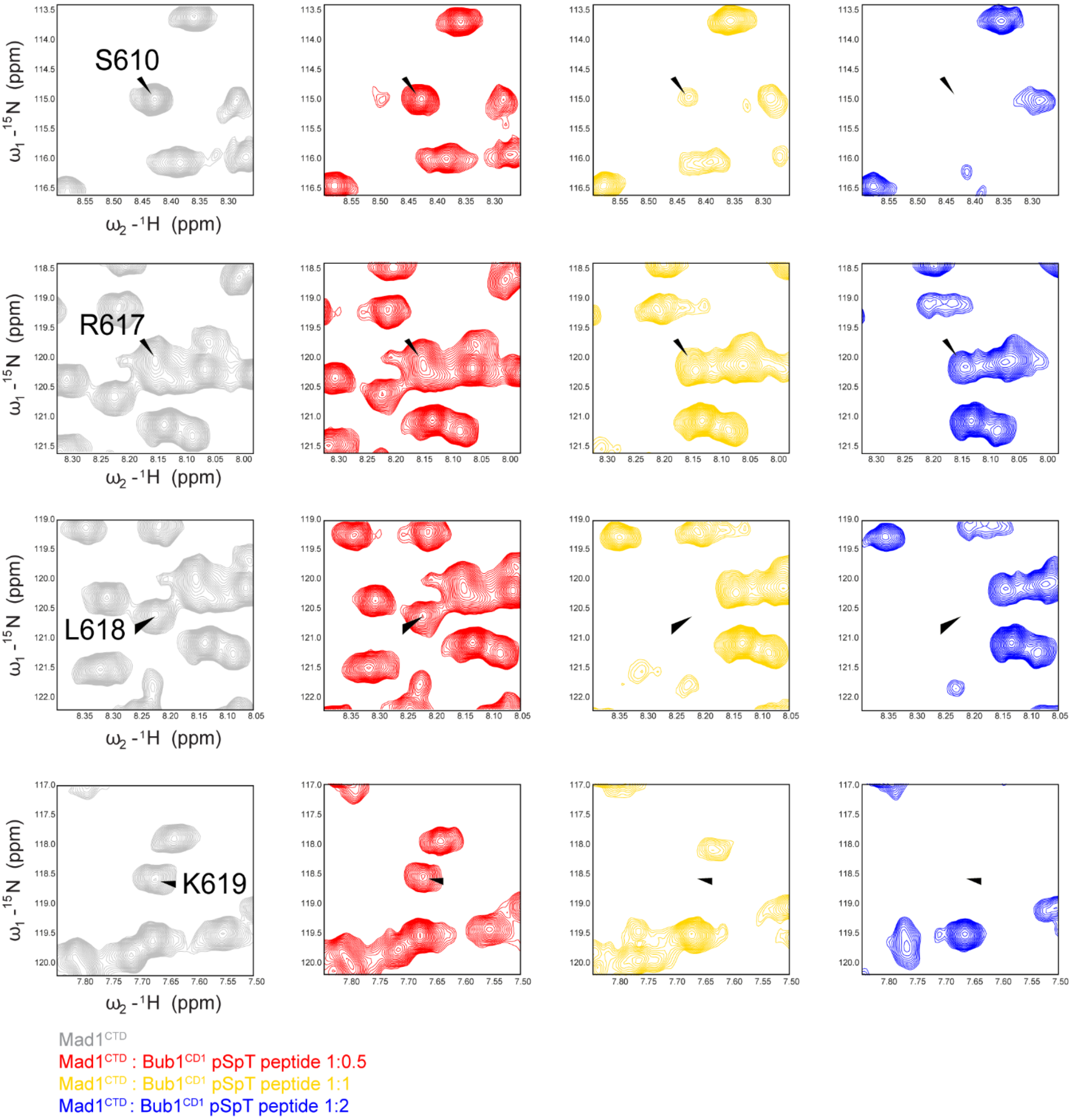

Appendix Fig S7: Close-up view of the backbone resonances of Mad1^{CTD} RLK motif residues upon Bub1^{CD1} peptide titration.

Close-up view in ^{1}H,^{15}N-2D HSQC showing ^{15}N-labelled Mad1^{CTD} with an increasing concentration of phosphorylated Bub1^{CD1} peptide. In molar ratios of Mad1^{CTD} dimer to Bub1^{CD1}, peptides were added at 1:0.5 (red), 1:1 (yellow) and 1:2 (blue). Resonances for Ser610, Arg617, Leu618 and Lys619 showed substantial attenuation as Bub1 peptides were titrated in.

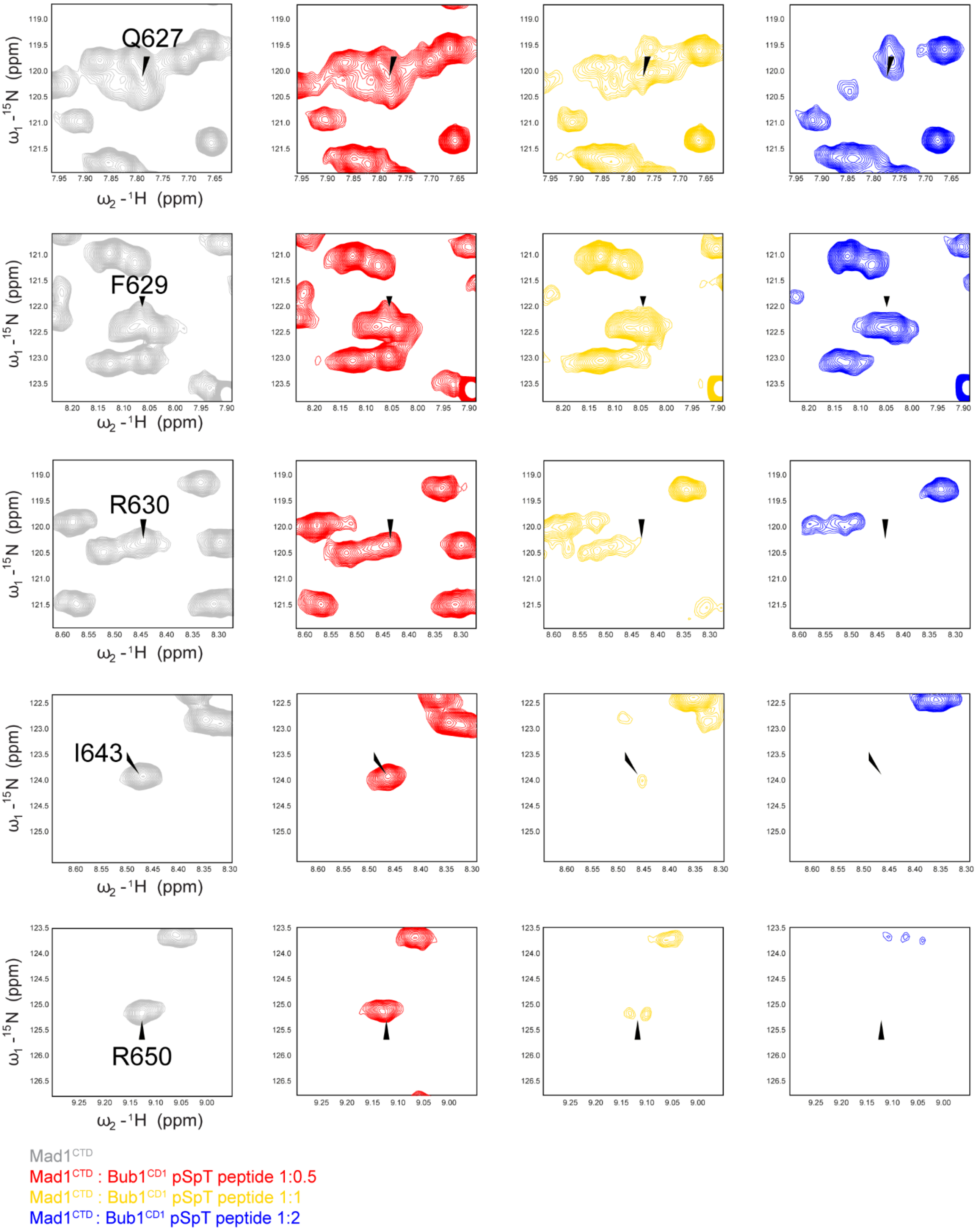

Appendix Fig S8: Close-up view of the backbone resonances of specific Mad1^{CTD} head domain residues upon Bub1^{CD1} peptide titration.
Close-up view in ^{1}H,^{15}N-2D HSQC showing ^{15}N-labelled Mad1^{CTD} with an increasing concentration of phosphorylated Bub1^{CD1} peptide. In molar ratios of Mad1^{CTD} dimer to Bub1^{CD1}, peptides were added at 1:0.5 (red), 1:1 (yellow) and 1:2 (blue). Significant line broadening was also observed for resonances corresponding to residues in the head domain Gln627, Phe629, Arg630, Ile643 and Arg650.

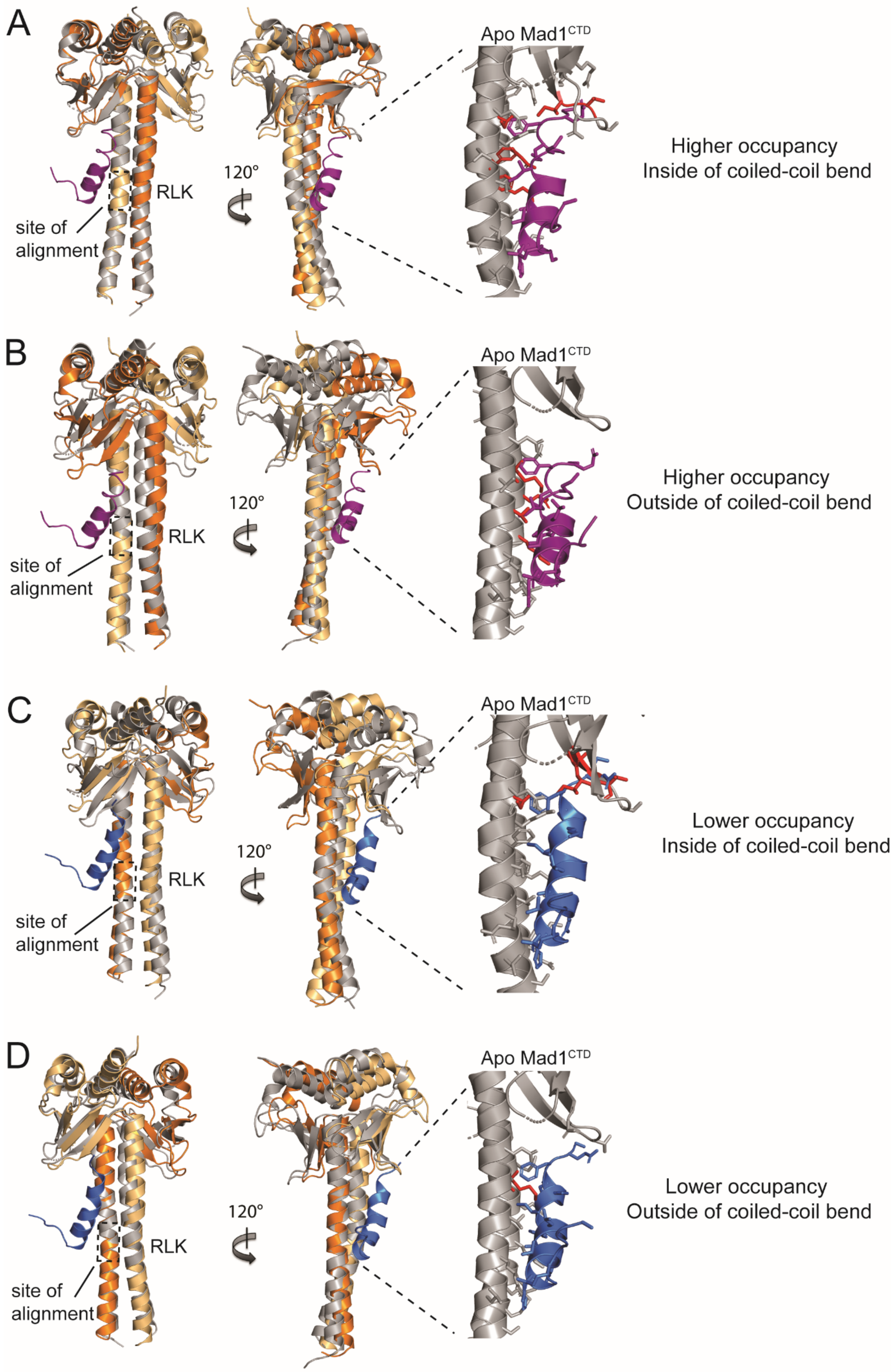
A
Apo Mad1CTD
Higher occupancy
Inside of coiled-coil bend
120°
RLK
site of
alignment
B
Apo Mad1CTD
Higher occupancy
Outside of coiled-coil bend
120°
RLK
site of
alignment
C
Apo Mad1CTD
Lower occupancy
Inside of coiled-coil bend
120°
RLK
site of
alignment
D
Apo Mad1CTD
Lower occupancy
Outside of coiled-coil bend
120°
RLK
site of
alignment

Appendix Fig S9: Alignments of apo and bound Mad1^{CTD}.

The various subunits of apo and bound Mad1^{CTD} are aligned by means of their RLK sites and the respective peptide bound is shown. For each alignment a close-up view of how the peptide fits onto the apo is shown, with clashes highlighted by residues coloured red. Bound and apo Mad1^{CTD} are coloured orange and grey correspondingly. The higher and lower occupancy peptides are coloured purple and blue correspondingly.

- A The RLK site of subunit B of bound Mad1^{CTD} (bound to the higher occupancy peptide) aligned onto the RLK site of subunit A of apo Mad1^{CTD}. Severe clashes occur with the coiled-coil and head.
- B The RLK site of subunit B of bound Mad1^{CTD} (bound to the higher occupancy peptide) aligned onto the RLK site of subunit in B of apo Mad1^{CTD}. Severe clashes occur with the coiled-coil and loss of contact with the head.
- C The RLK site of subunit A of bound Mad1^{CTD} (bound to the lower occupancy peptide) aligned onto the RLK site of subunit A of apo Mad1^{CTD}. Severe clashes occur with the head.
- D The RLK site of subunit A of bound Mad1^{CTD} (bound to the lower occupancy peptide) aligned onto the RLK site of subunit B of apo Mad1^{CTD}. Slight clashes occur with the coiled-coil and partial loss of contact with the head.
