# Supplementary material for: Molecular mechanism of Mad1 kinetochore targeting by phosphorylated Bub1
Source: EMBO Rep. 2021 May 19;22(7):e52242. doi: 10.15252/embr.202052242 (PMC8391104; doi:10.15252/embr.202052242)
Supplement: Supplementary file 2 — Expanded View Figures PDF [file EMBR-22-e52242-s003.pdf]

Expanded View Figures

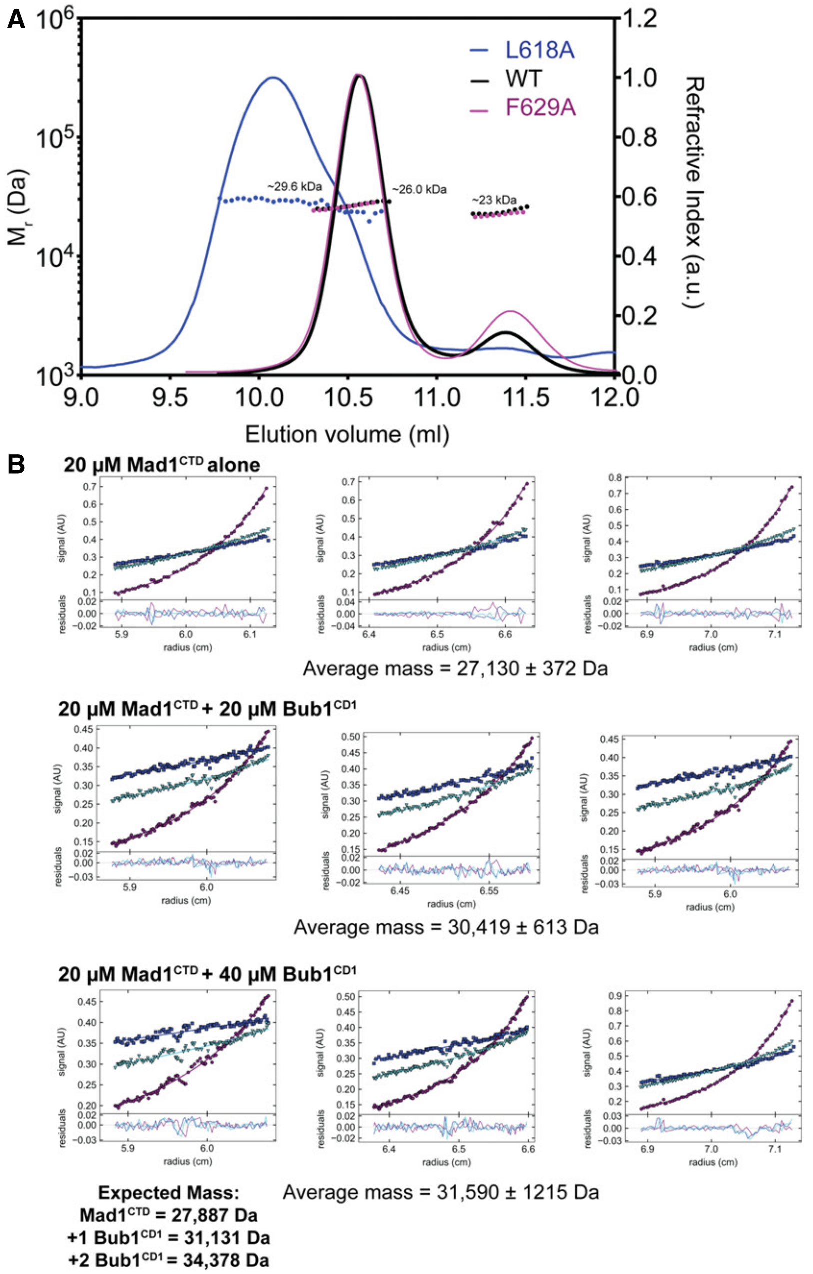

Figure EV1. Biophysical analysis of Mad1^{CTD} complexes.

A Size-exclusion multi-angle light scattering (SEC-MALS) of Mad1^{CTD} wild-type and L618A and F629A mutants. All eluted as monodispersed species. The average mass of WT Mad1^{CTD} was 26.2 kDa. The average mass for the L618A and F629A mutants was 29.6 and 26.7 kDa, respectively. A tailing peak of about 23 kDa most likely comes from residual amount of TEV protease in the sample.

B Analytical ultracentrifugation sedimentation equilibrium of Mad1^{CTD}-Bub1^{CD1} complexes. Samples were run in triplicate. The data were fitted to a two-species model where the mass of the Bub1^{CD1} peptide with an N-terminal tryptophan residue (3,247 Da) was fixed. Standard errors are shown.

© 2021 The Authors

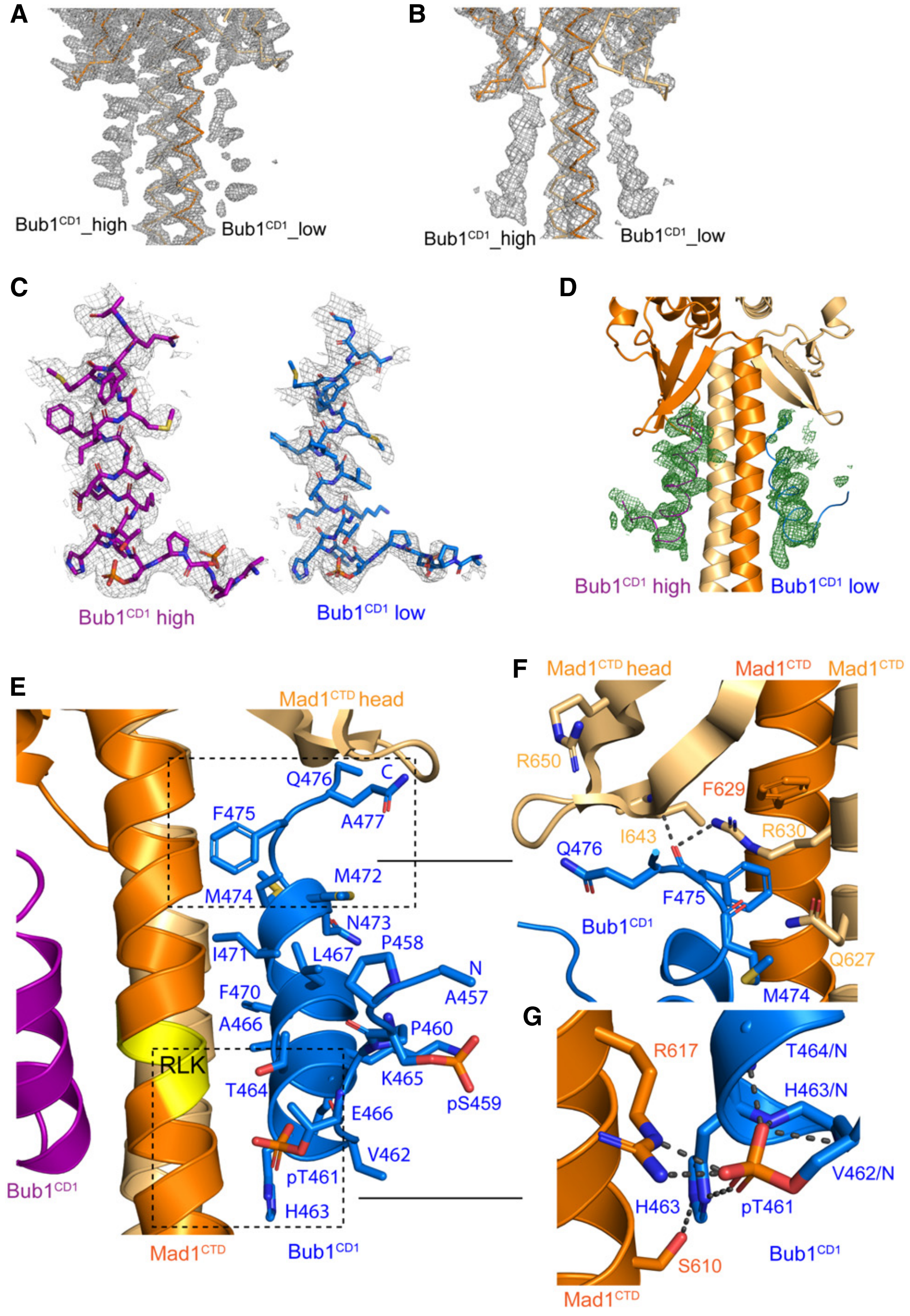

Figure EV2.

 © 2021 The Authors

◀ **Figure EV2. Differential peptide occupancy in the Mad1^{CTD}-Bub1^{CD1} crystal structure.**

A A snapshot of the electron density map of Mad1^{CTD}-Bub1^{CD1} visualized in Coot (Emsley *et al*, 2010) which shows the differential Bub1^{CD1} peptide occupancy. Both Mad1^{CTD} subunits are depicted as ribbons in shades of blue. Bub1^{CD1}_high has higher occupancy and is represented as purple throughout this paper. Bub1^{CD1}_low has lower occupancy and is represented as blue.

B Electron density map visualized in Coot for a lower resolution Mad1^{CTD}-Bub1^{CD1} structure showing more equivalent peptide occupancy (Emsley *et al*, 2010). Two times the peptide concentration as compared to the complex crystallized in (A) was used during co-crystallization (5 mM total) requiring 10% DMSO.

C Electron density map for each peptide is shown using Isomesh in PYMOL. The same threshold for each peptide is displayed. Differences between the sidechain density and positioning of each peptide can be seen, as well as clear phosphate density for both pThr461 phosphates.

D The 2Fo-Fc omit map (green) for both Bub1^{CD1} peptides in the Bub1^{CD1}-Mad1^{CTD} crystal structure. Created with Phenix (Liebschner *et al*, 2019).

E The extensive interface of the Mad1^{CTD}-Bub1^{CD1} interaction is highlighted with the lower occupancy Bub1^{CD1} peptide (blue). The RLK motif of Mad1^{CTD} is coloured yellow.

F Close-up view of the lower occupancy Bub1^{CD1} peptide (blue) interactions with the head domain of Mad1^{CTD} (orange/light orange). Hydrogen bonding interactions within 3.5 Å are highlighted by black dashes.

G Close-up view of the Mad1^{CTD} Arg617 and Bub1^{CD1} pThr461 interaction in the lower occupancy peptide (blue). Hydrogen bonding interactions within 3.5 Å are highlighted by black dashes. Additional contact occurs between the phosphate of pThr461 and Bub1^{CD1} His463 which then forms a hydrogen bond with Mad1^{CTD} Ser610. Additional stabilizing hydrogen bonding occurs between the pThr461 phosphate and the amide nitrogen of Val462, His463 and Thr464.

© 2021 The Authors

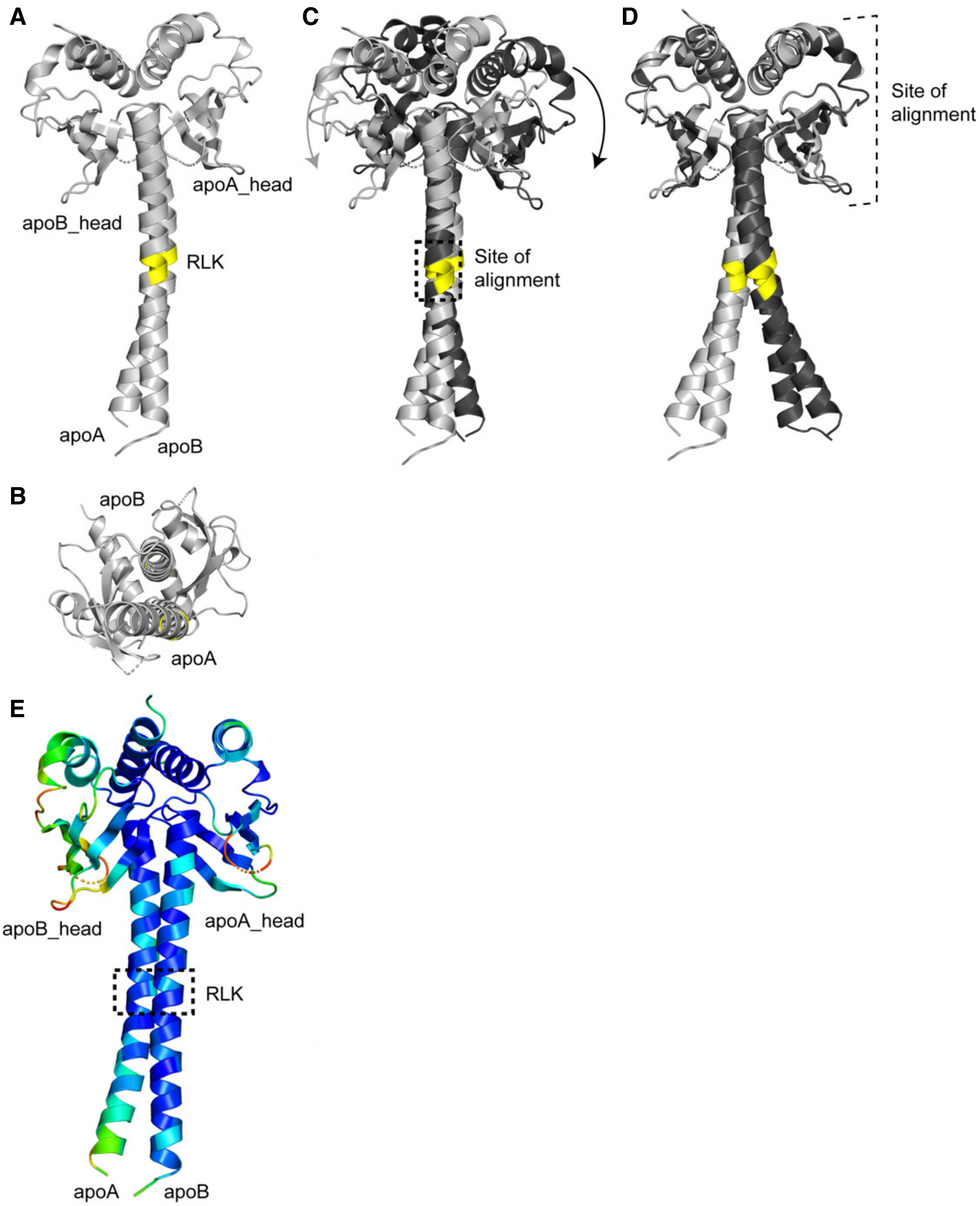

Figure EV3. Apo Mad1^{CTD} homodimer is asymmetric.

A Crystal structure of apo Mad1^{CTD} (grey) with the RLK motif highlighted in yellow. PDB ID: 4DZO (Kim *et al*, 2012).
B Bottom view of the crystal structure of apo Mad1^{CTD} showing the extent of the bending of apoA helix.
C Opposite subunits of two copies of apo Mad1^{CTD} homodimer aligned onto their respective RLK motifs (yellow). One copy is in grey, the other in black. The arrows highlight the rotation of the head domain inwards towards the inside of the coiled-coil curvature.
D Alignment of opposite subunits of the head domain of two apo Mad1^{CTD} copies. One copy is in grey, the other in black.
E Apo Mad1^{CTD} coloured by relative B-factors. The more bent helix (apoA) and its adjacent head domain (apoB_head) exhibit higher flexibility.

Source data are available online for this figure.

 © 2021 The Authors

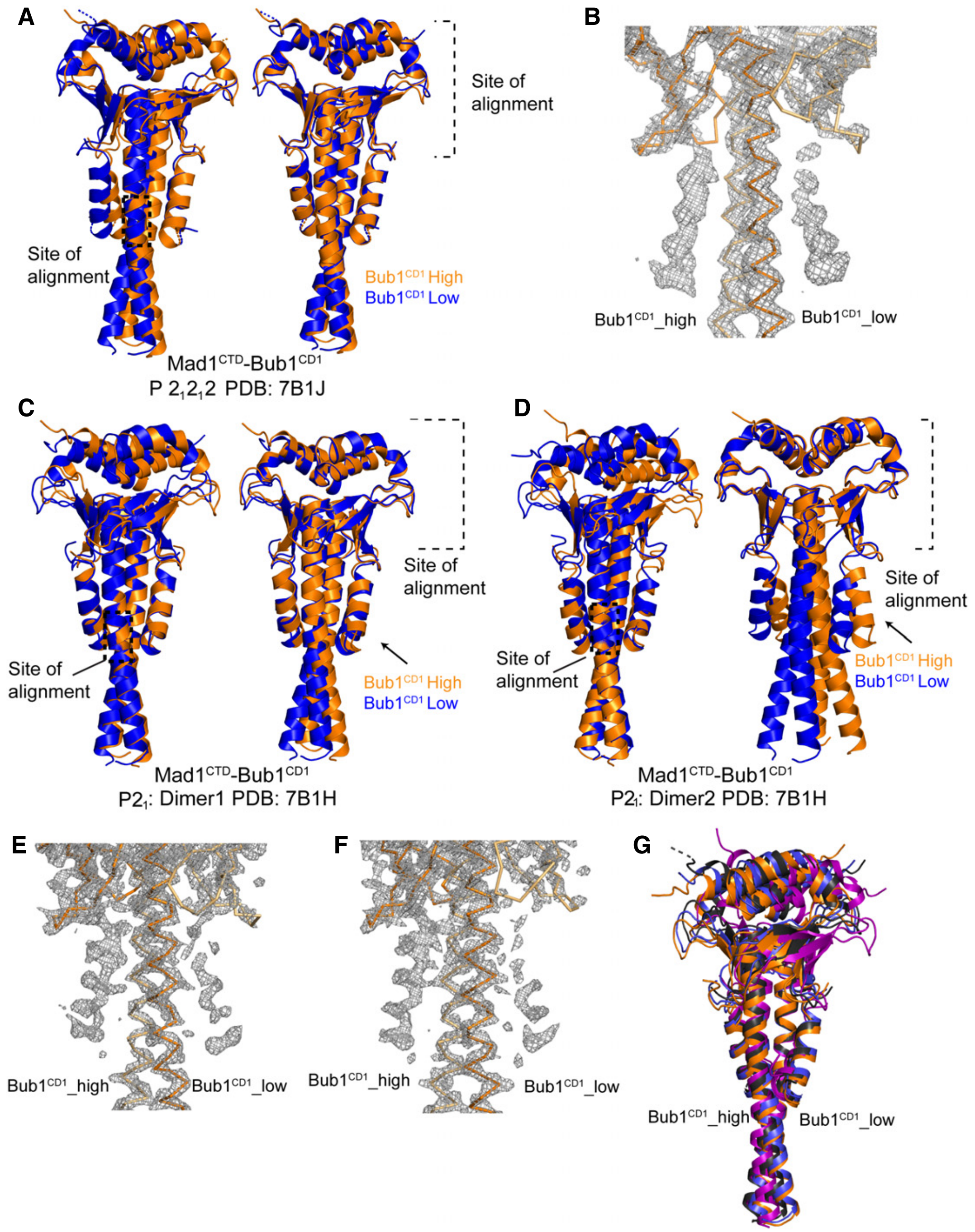
A
Site of alignment
Site of alignment
Bub1CD1 High
Bub1CD1 Low
Mad1CTD-Bub1CD1
P 2₁2₁2 PDB: 7B1J
B
Bub1CD1_high
Bub1CD1_low
C
Site of alignment
Site of alignment
Bub1CD1 High
Bub1CD1 Low
Mad1CTD-Bub1CD1
P2₁: Dimer1 PDB: 7B1H
D
Site of alignment
Site of alignment
Bub1CD1 High
Bub1CD1 Low
Mad1CTD-Bub1CD1
P2₁: Dimer2 PDB: 7B1H
E
Bub1CD1_high
Bub1CD1_low
F
Bub1CD1_high
Bub1CD1_low
G
Bub1CD1_high
Bub1CD1_low

Figure EV4.

◀ **Figure EV4. Mad1^{CTD}-Bub1^{CD1} asymmetry and differential Bub1^{CD1} occupancy are conserved across structures from different space groups.**

A Alignment of Mad1^{CTD}-Bub1^{CD1} structure from the $P2_12_12$ space group (PDB: 7B1J). Left panel: Alignment of opposite subunits of the homodimer on the RLK site. Right panel: Alignment of opposite subunits on the head domain. The duplicated dimers are coloured in orange or blue.

B Electron density map of the Mad1^{CTD}-Bub1^{CD1} structure from the $P2_12_12$ space group (PDB: 7B1J) visualized in Coot (Emsley *et al*, 2010) which shows near equivalent peptide occupancy. The Mad1^{CTD} homodimer is represented as a ribbon in orange and yellow.

C Alignment of one homodimer from the Mad1^{CTD}-Bub1^{CD1} structure from the $P2_1$ space group (PDB: 7B1H). Left panel: Alignment of opposite subunits of the homodimer on the RLK site. Right panel: Alignment of opposite subunits on the head domain. The duplicated dimers are coloured in orange or blue.

D Alignment of the second homodimer from the Mad1^{CTD}-Bub1^{CD1} structure from the $P2_1$ space group (PDB: 7B1H). Left panel: Alignment of opposite subunits of the homodimer on the RLK site. Right panel: Alignment of opposite subunits on the head domain. The duplicated dimers are coloured in orange or blue.

E Electron density map of one homodimer from the Mad1^{CTD}-Bub1^{CD1} structure (shown directly above) from $P2_1$ space group visualized in Coot (Emsley *et al*, 2010). The Mad1^{CTD} homodimer is represented as a ribbon in green and yellow.

F Electron density map of the second homodimer from the Mad1^{CTD}-Bub1^{CD1} structure (shown directly above) from the $P2_1$ space group visualized in Coot (Emsley *et al*, 2010). The Mad1^{CTD} homodimer is represented as a ribbon in pink and blue. The occupancy of the peptide on the right is extremely poor as is the density for the head domain it contacts.

G All four Mad1^{CTD} homodimers from the three different space group structures are aligned onto their Mad1^{CTD} RLK motif which is bound to the higher occupancy peptide. Blue = homodimer_1 from $P2_1$. Orange = homodimer_2 from $P2_1$. Purple = $P2_12_12_1$ homodimer. Black = $P2_12_12$.

Source data are available online for this figure.

 © 2021 The Authors

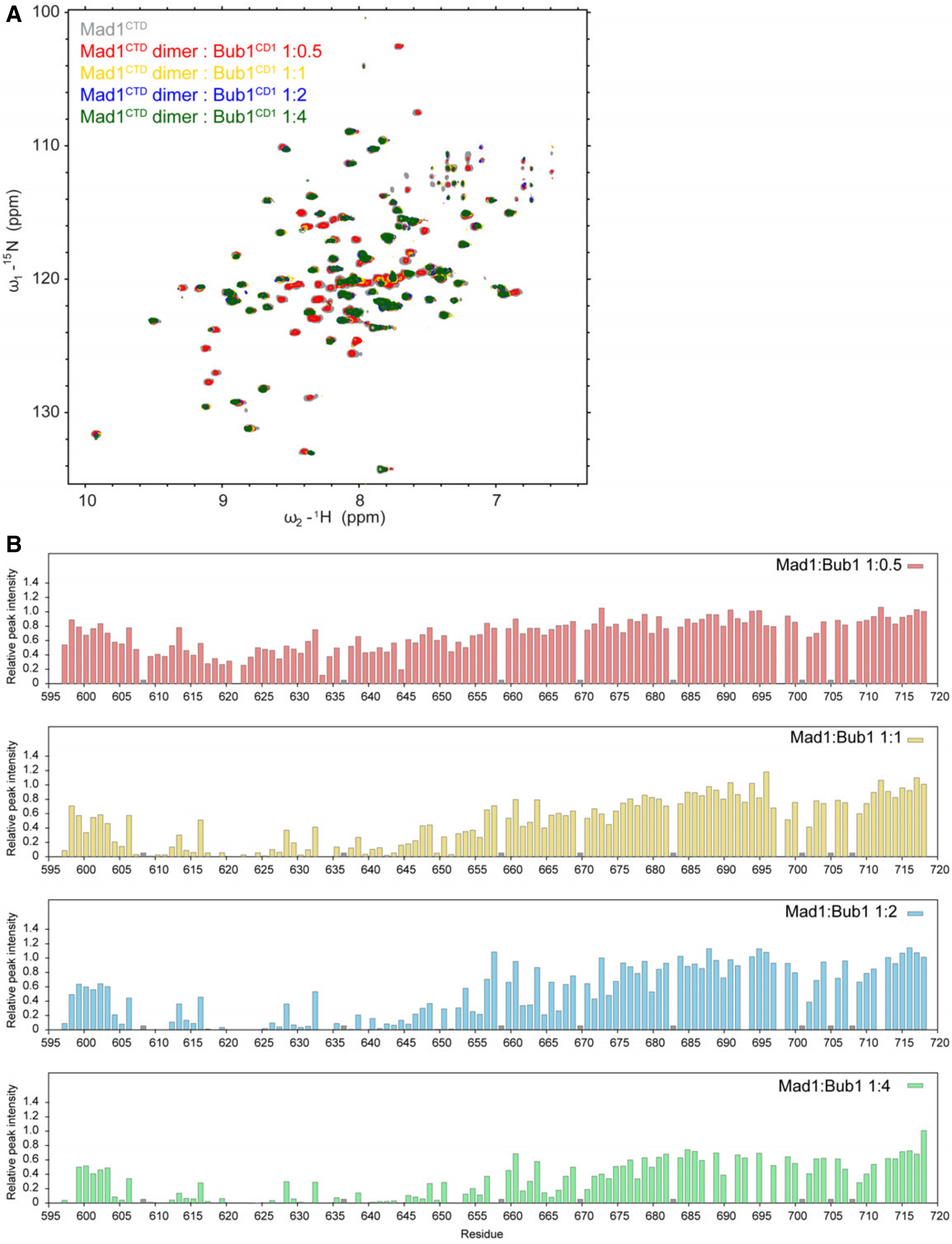

Figure EV5. Titration of phosphorylated Bub1^{CD1} peptide into Mad1^{CTD}.

A ^{1}H,^{15}N-2D HSQC showing ^{15}N-labelled Mad1^{CTD} with an increasing concentration of phosphorylated Bub1^{CD1} peptide. In molar ratios of Mad1^{CTD} dimer to Bub1^{CD1} peptides were added at 1:0.5 (red), 1:1 (yellow), 1:2 (blue) and 1:4 (green) ratios.

B Relative peak intensities of Mad1^{CTD} upon titration of phosphorylated Bub1^{CD1} peptides. The bar charts follow a similar colour scheme as the spectra in A, with molar ratios of Mad1^{CTD} dimer to Bub1^{CD1} at 1:0.5 (red), 1:1 (yellow), 1:2 (blue) and 1:4 (green) ratios. Peak intensities were normalized to that of the C-terminal residue Ala718.

Source data are available online for this figure.

© 2021 The Authors
